# Application of artificial intelligence in modern healthcare for diagnosis of autism spectrum disorder

**DOI:** 10.3389/fmed.2025.1569464

**Published:** 2025-05-21

**Authors:** Abdullah H. Al-Nefaie, Theyazn H. H. Aldhyani, Sultan Ahmad, Eidah M. Alzahrani

**Affiliations:** ^1^King Salman Center for Disability Research, Riyadh, Saudi Arabia; ^2^Department of Quantitative Methods, School of Business, King Faisal University, Hofuf, Saudi Arabia; ^3^Applied College in Abqaiq, King Faisal University, Hofuf, Saudi Arabia; ^4^Department of Computer Science, College of Computer Engineering and Sciences, Prince Sattam Bin Abdulaziz University, Al-Kharj, Saudi Arabia; ^5^Computer Science Department, Al-Baha University, Al-Bahah, Saudi Arabia

**Keywords:** transfer learning, deep learning, diagnosis, disability, mental health

## Abstract

**Introduction:**

Symptoms of autism spectrum disorder (ASD) range from mild to severe and are evident in early childhood. Children with ASD have difficulties with social interaction, language development, and behavioral regulation. ASD is a mental condition characterized by challenges in communication, restricted behaviors, difficulties with speech, non-verbal interaction, and distinctive facial features in children. The early diagnosis of ASD depends on identifying anomalies in facial function, which may be minimal or missing in the first stages of the disorder. Due to the unique behavioral patterns shown by children with ASD, facial expression analysis has become an effective method for the early identification of ASD.

**Methods:**

Hence, utilizing deep learning (DL) methodologies presents an excellent opportunity for improving diagnostic precision and efficacy. This study examines the effectiveness of DL algorithms in differentiating persons with ASD from those without, using a comprehensive dataset that includes images of children and ASD-related diagnostic categories. In this research, ResNet50, Inception-V3, and VGG-19 models were used to identify autism based on the facial traits of children. The assessment of these models used a dataset obtained from Kaggle, consisting of 2,940 face images.

**Results:**

The suggested Inception-V3 model surpassed current transfer learning algorithms, achieving a 98% accuracy rate.

**Discussion:**

Regarding performance assessment, the suggested technique demonstrated advantages over the latest models. Our methodology enables healthcare physicians to verify the first screening for ASDs in children.

## 1 Introduction

**Figure 1:**
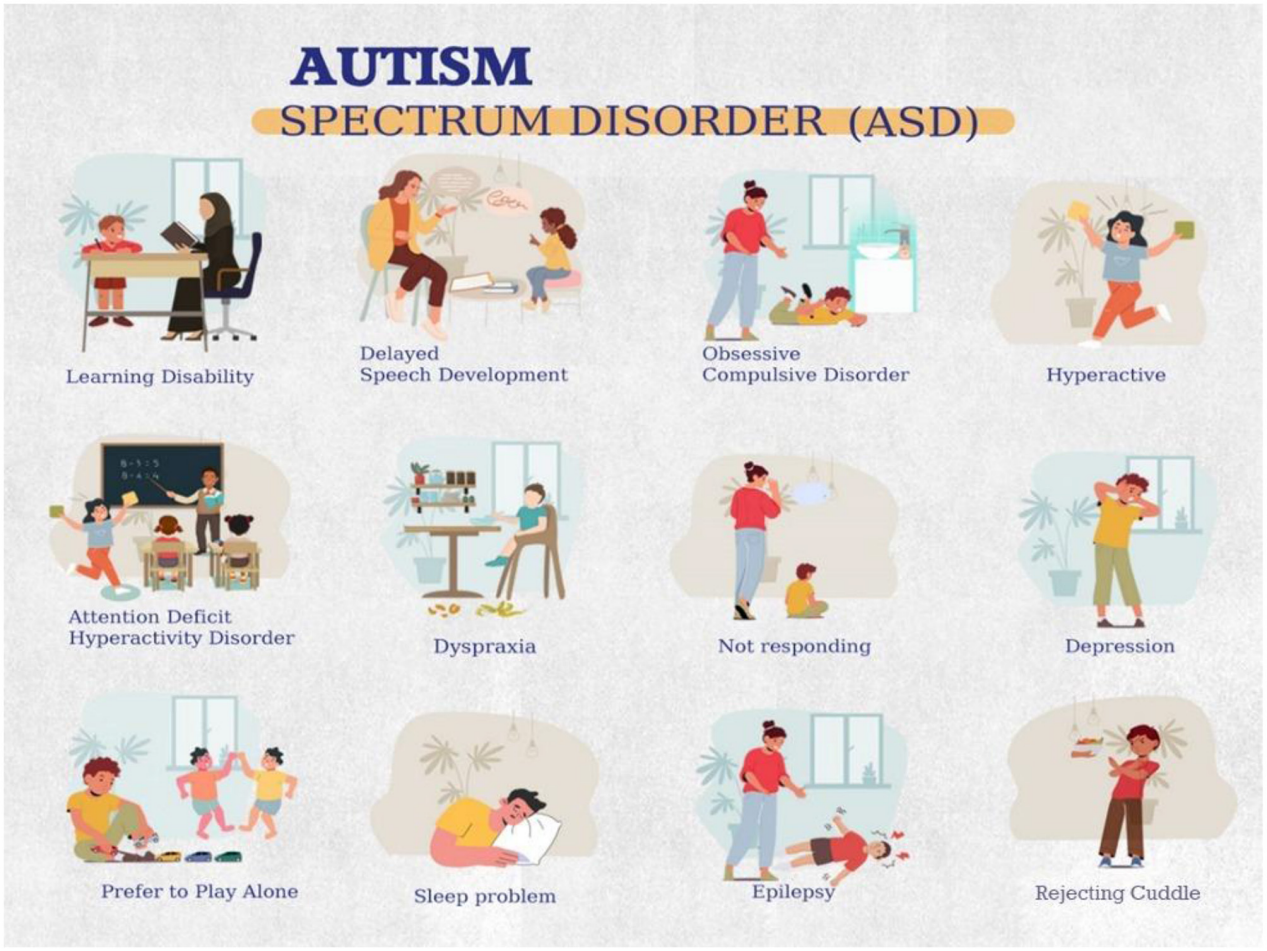
Symptoms of ASD.

Autism Spectrum Disorder (ASD) represents one of the most significant challenges in modern neurodevelopmental medicine, affecting ~1 in 36 children globally (1). This complex condition, characterized by difficulties in social interaction, communication patterns, and repetitive behaviors, demands early intervention for optimal outcomes (2). ASD is identified based on deficiencies in behavioral skills and social communication, often seen via recurrent behavioral indicators in children. Figure 1 displays the symptoms of ASD. However, traditional diagnostic procedures usually involve time-intensive behavioral assessments and costly medical evaluations, creating substantial barriers to early detection, particularly in resource-limited settings (3).

Recent advances in artificial intelligence, particularly in the domain of deep learning and computer vision, have opened promising new avenues for ASD screening (4, 5). The emerging field of facial phenotype analysis is of particular interest, which leverages the observation that individuals with ASD often present distinct facial morphological characteristics (6). These features, including broader upper faces, wider eyes, shorter nasal bridges, and narrower cheeks, have been increasingly recognized as potential biomarkers for ASD detection (6).

Timely diagnosis facilitates the use of specialist therapies designed to address the unique requirements of persons with autism, focusing on social communication, language development, and behavioral issues. Moreover, early diagnosis allows families to get suitable support services, educational resources, and community activities, enhancing coping strategies, alleviating parental stress, and promoting adult independence.

Nonetheless, early identification of autism by traditional methods also has specific threats. A significant concern is the potential for labeling, which may impact the child's self-esteem and social relationships. There is a risk of overdiagnosis or misdiagnosis, leading to unnecessary interventions and therapies. The diagnostic procedure may be delayed, intricate, and emotionally testing for families, necessitating thorough evaluations by multidisciplinary teams. Consequently, using sophisticated approaches supported by artificial intelligence (AI) may mitigate this danger, as AI utilizes technology capable of incorporating feedback from youngsters, informed by their expertise. In this study, we used facial images of children to identify those suffering from ASD.

The integration of deep learning methodologies with facial analysis represents a potentially transformative approach to ASD screening. Contemporary deep learning architectures have demonstrated remarkable capabilities in extracting complex patterns from facial images, offering the possibility of automated, rapid, and cost-effective screening tools. This approach aligns with the growing need for accessible screening methods that can support healthcare professionals in identifying individuals who may require comprehensive diagnostic evaluation.

This research presents a novel deep learning framework for ASD detection through facial image analysis. Our study evaluates the performance of three state-of-the-art deep learning architectures: ResNet, VGG16, and VGG19. Through rigorous experimentation and validation, we demonstrate that the VGG19 architecture achieves superior performance with an accuracy of 98%, representing a significant advancement in automated ASD screening capabilities.

The primary contributions of this study include:

1. A comprehensive evaluation of DL architectures for facial image-based ASD detection.
2. The development of an optimized VGG19-based model achieving 98% accuracy.
3. Analysis of the specific facial features that contribute most significantly to accurate ASD detection.

This research aims to advance the field of automated ASD screening, potentially reducing the burden on healthcare systems while accelerating the identification of individuals who may benefit from early intervention. Our findings suggest that deep learning-based facial analysis could serve as a valuable complementary tool in the ASD diagnostic process, particularly in settings where access to traditional diagnostic resources is limited.

The research gap in ASD identification using images persists, despite the proposed system achieving 98% accuracy on a benchmark dataset. Different signals in facial expressions make it challenging to identify using advanced deep learning models, which may aid in predicting ASD. Ultimately, clinical validation is necessary to ensure the widespread adoption of this approach in healthcare settings and its practical applicability.

## 2 Related work

Early detection of ASD is crucial for effective intervention and treatment (7). While traditional diagnostic methods rely on clinical observations and behavioral assessments such as the Autism Diagnostic Observation Schedule (ADOS) (8), recent years have seen significant advancement in automated detection approaches. These advancements span multiple modalities, including facial analysis (9), magnetic resonance imaging (MRI) (10), eye tracking (11, 12), and electroencephalography (EEG) (13). The emergence of sophisticated machine learning and deep learning techniques has particularly accelerated the development of automated diagnostic systems across these modalities (13), offering promising tools for early screening and detection.

Akter et al. (14) conducted work using transfer learning, working with a dataset of 2,936 facial images from Kaggle. Their study evaluated multiple machine learning classifiers and pre-trained CNN models, with their improved MobileNet-V1 model achieving an accuracy of 90.67%. They used K-means clustering to identify potential ASD subtypes, achieving 92.10% accuracy for two autism subtypes. Elshoky et al. (15) comprehensively compared machine learning approaches using facial images from Kaggle. Their study uniquely compared classical machine learning, deep learning, and automated machine learning (AutoML) approaches. Using OpenCV for pre-processing with 90 × 90 pixel resizing and grayscale conversion, their AutoML approach achieved ~96% accuracy, significantly outperforming classical ML 72.64% with Extra Trees and deep learning methods using VGG16, which achieved 89%.

Li et al. (16) introduced a two-phase transfer learning approach using MobileNetV2 and MobileNetV3-Large. Their method transferred knowledge from ImageNet to facial images from Kaggle. This mobile-optimized approach achieved 90.5% accuracy with an AUC of 96.32%. Siagian et al. (17) took a different approach, using a unique dataset of 200 facial images collected from special schools in Medan, Indonesia. Their method combined the SURF (Speeded-Up Robust Features) algorithm with various boosting methods, achieving 91.67% accuracy with Gradient Boosting despite the relatively small dataset.

Alkahtani et al. (18) explored a hybrid approach combining pre-trained CNNs with traditional machine learning classifiers. Their study utilized MobileNetV2 and VGG19 as feature extractors, paired with various classifiers machine learning algorithms. Working with a publicly available dataset, their optimized MobileNetV2 configuration, using the Adamax optimizer with a learning rate of 0.001, achieved 92% accuracy. Sai Koppula and Agrawal (19) evaluated multiple pre-trained CNN architectures with a focus on domain-specific variations. Using the Kaggle dataset, they implemented extensive data augmentation through Keras' ImageDataGenerator. Their study revealed that models pre-trained on VGGFace2 outperformed those trained on ImageNet, with VGG16 achieving 86% accuracy and AUC. Abdullah et al. (20) explored an ensemble approach that combined the EfficientNet B5, MobileNet, and InceptionV3 models using the Kaggle dataset. Their method employed data augmentation techniques and utilized a soft voting ensemble method, achieving an accuracy of 89.87%. Karthik et al. (21) investigated hybrid deep learning models using Vision Transformers (ViT) with various classifiers. Working with the Kaggle dataset, they implemented comprehensive pre-processing, including grayscale conversion, resizing to 224 × 224 pixels, normalization, and extensive augmentation. Their ViT model, combined with XGBoost and SHAP implementation, achieved 91.3% accuracy.

Pan and Foroughi (22) focused on edge computing applications, adapting AlexNet for efficient processing in educational environments using the Kaggle dataset. Their implementation achieved 93.24% accuracy while maintaining real-time processing capabilities, demonstrating the feasibility of edge deployment for ASD screening tools. Shahzad et al. (23) introduced a hybrid attention-based model combining ResNet101 and EfficientNetB3. Their approach incorporated self-attention mechanisms from natural language processing and emphasized standardized pre-processing with image augmentation through rotations, zooming, and flipping. The hybrid attention-based model achieved an accuracy of 96.50%. Reddy and Andrew (24) conducted a comparative study of three pre-trained Convolutional Neural Network (CNN) architectures: VGG16, VGG19, and EfficientNetB0. Their investigation utilized a dataset of facial images of children, implementing comprehensive data augmentation techniques, including rotation, horizontal flipping, zooming, and height/width shifting. Images were standardized to 227 × 227 × 3 pixels to ensure compatibility with the CNN architectures. Their findings revealed that EfficientNetB0 achieved the highest accuracy at 87.9%, surpassing both VGG16 84.66% and VGG19 80.05%. Table 1 displays the different existing systems that have been developed for the diagnosis of ASD.

**Table 1:** Existing using facial images.

**Study**	**Dataset**	**Methods/models**	**Key findings/accuracy**
Akter et al. (2021) (14)	Autism Face Image Dataset	Transfer learning with MobileNet-V1, K-means clustering	MobileNet-V1: 90.67%; Clustering: 92.10% for ASD subtypes
Elshoky et al. (2022) (15)	Autism Face Image Dataset	Classical ML, Deep Learning (VGG16), AutoML	AutoML: 96%; VGG16: 89%; Classical ML (Extra Trees): 72.64%
Li et al. (2023) (16)	Autism Face Image Dataset	Two-phase transfer learning	MobileNetV3-Large: 90.5%, AUC: 96.32%
Siagian et al. (2023) (17)	Special dataset of 200 images	Gradient Boosting with SURF features	Gradient Boosting: 91.67%
Alkahtani et al. (2023) (18)	Autism Face Image Dataset	MobileNetV2, VGG19 with various classifiers	MobileNetV2: 92%
Sai Koppula and Agrawal (2023) (19)	Autism Face Image Dataset	VGGFace2 vs. ImageNet-based pre-trained CNNs	VGG16 (VGGFace2): 86%, AUC: Not specified
Abdullah et al. (2024) (20)	Autism Face Image Dataset	Ensemble (EfficientNetB5, MobileNet, InceptionV3)	Ensemble: 89.87%
Karthik et al. (2024) (21)	Autism Face Image Dataset	Vision Transformers (ViT) with XGBoost and SHAP	ViT + XGBoost: 91.3%
Pan and Foroughi (2024) (22)	Autism Face Image Dataset	Edge-optimized AlexNet	AlexNet: 93.24%
Shahzad et al. (2024) (23)	Autism Face Image Dataset	ResNet101 + EfficientNetB3 hybrid with self-attention	Hybrid: 96.50%
Reddy and Andrew (2024) (24)	Autism Face Image Dataset	VGG16, VGG19, EfficientNetB0	EfficientNetB0: 87.9%; VGG16: 84.66%; VGG19: 80.05%

## 3 Materials and methods

**Figure 2:**
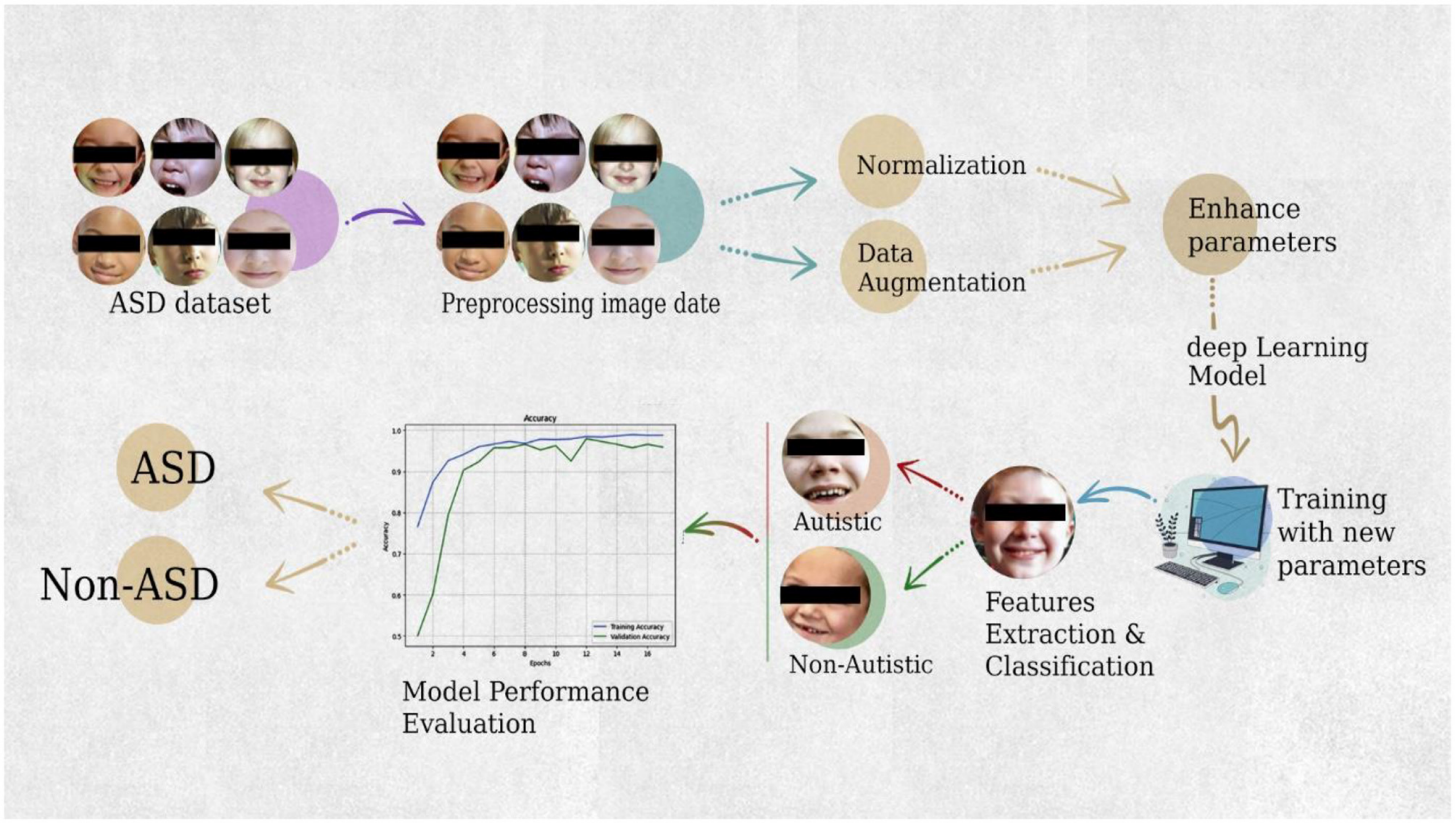
Enhanced diagnosis ASD system.

This research used DL models to predict and classify ASD in children at an early stage. This framework was developed using autistic face features. This study used pre-trained DL models to automatically extract robust characteristics of children's faces to detect ASD. The framework of the proposed ASD system is presented in Figure 2.

### 3.1 Dataset

**Figure 3:**
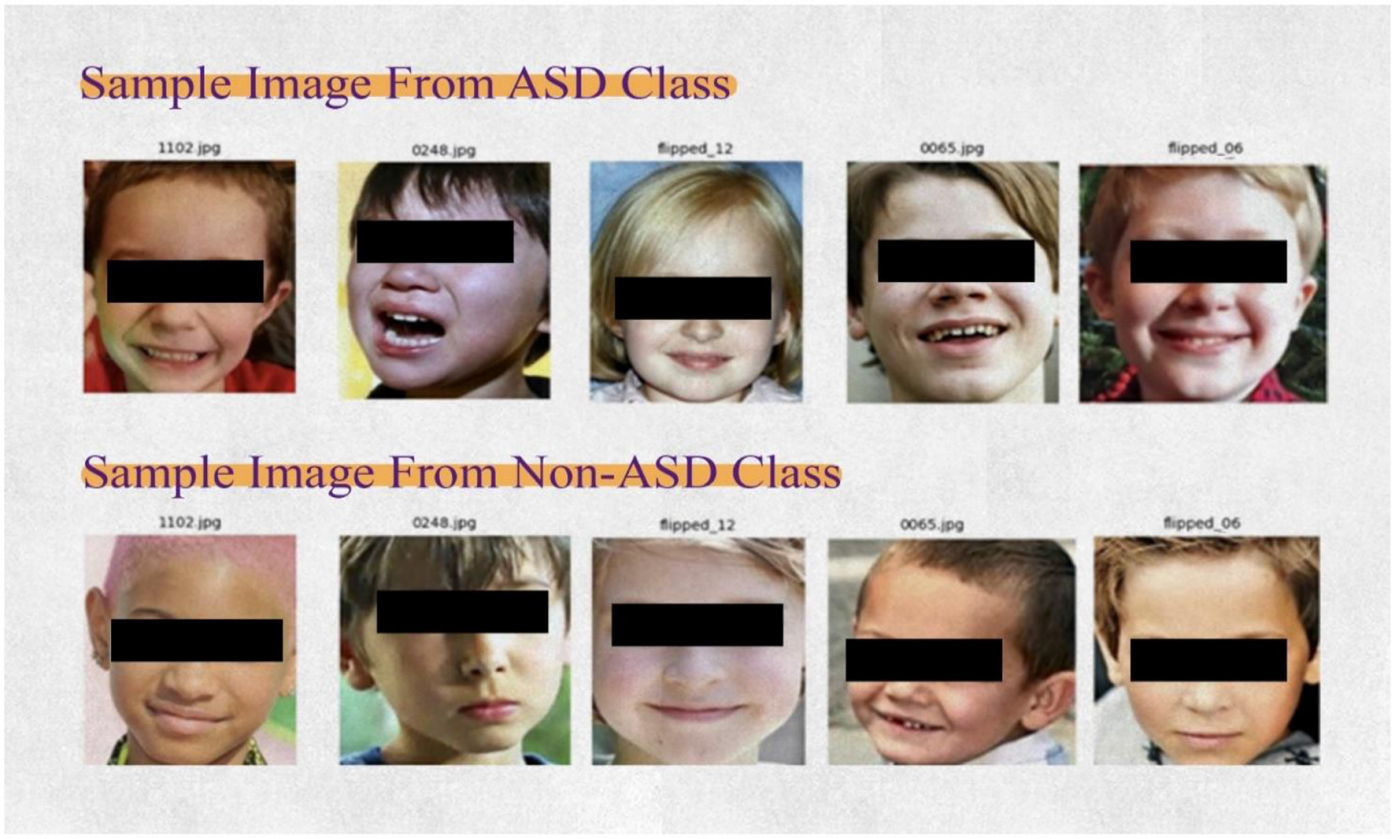
Snapshot of dataset.

The research used face images of autistic children from a publicly accessible collection (Kaggle). The dataset included 2D RGB images of children aged 2–14. The dataset was designed into two subfolders: one designated for autistic children and the other for non-autistic children. The autistic subfolder included images of ASD, while the non-autistic subfolder had images randomly retrieved from web searches, as shown in Table 2. The images were sized at 224 × 224 × 3, providing a comparative overview of ASD and non-ASD images. The snapshots of images of ASD and non-ASD are presented in Figure 3.

**Table 2:** Samples of dataset.

**Dataset**	**Number**
Total_images	2,940
Autistic_children	1,327
Non-autistic_childern	1,613

### 3.2 Pre-processing approach

#### 3.2.1 Data augmentation

Data augmentation is process to generating additional data from existing datasets to train deep learning models, which might be complicated by data silos, restrictions, and other constraints, by minor modifications to the original data. This study employs data augmentation to enhance the model's efficacy by artificially expanding the training dataset by transformations such as flipping, shearing, zooming, and rescaling, as shown in Table 3. These parameters mitigate overfitting when the model retains training data rather than acquiring generalized patterns, thereby improving the model's efficacy. The ASD and Non-ASD images in standard collections may be constrained in size; augmentation artificially enhances them by rescaling pixel values to [0, 1], shearing images by 10%, zooming by 10%, and performing horizontal flipping.

**Table 3:** Augmentation parameters.

**Indicators**	**Values**
Shear_Range method	0.1
Zoom_Range method	0.1
Horizontal_Flip method	True

#### 3.2.2 Data splitting

**Figure 4:**
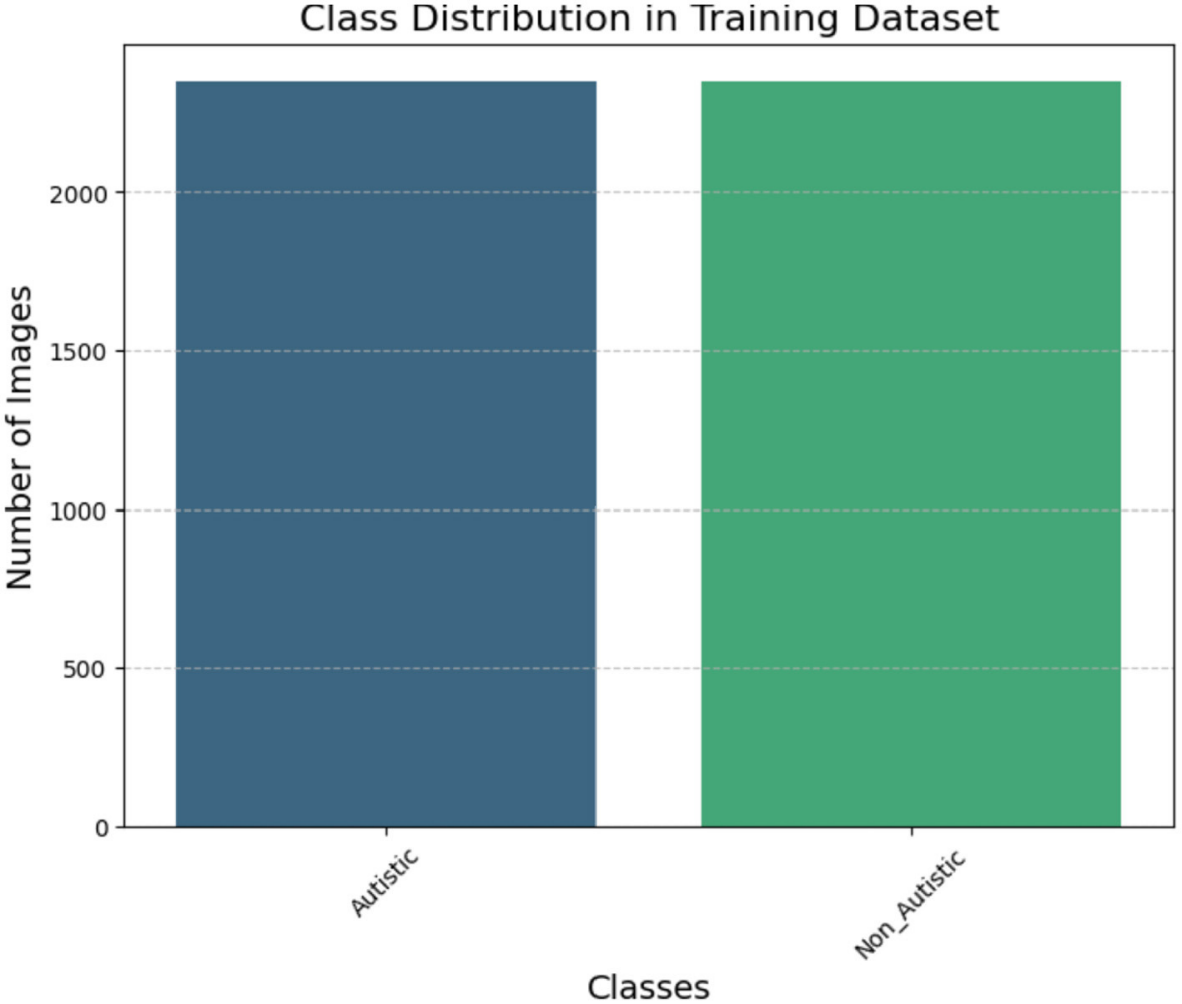
Class ASD dataset.

The dataset is partitioned into three sets: training (80%), validation (10%), and test (10%). This guarantees that the model is tested on unknown data for improved generalizability. The class volume of the ASD dataset is presented in Figure 4.

### 3.3 Deep learning models

#### 3.3.1 Inception-V3 models

**Figure 5:**
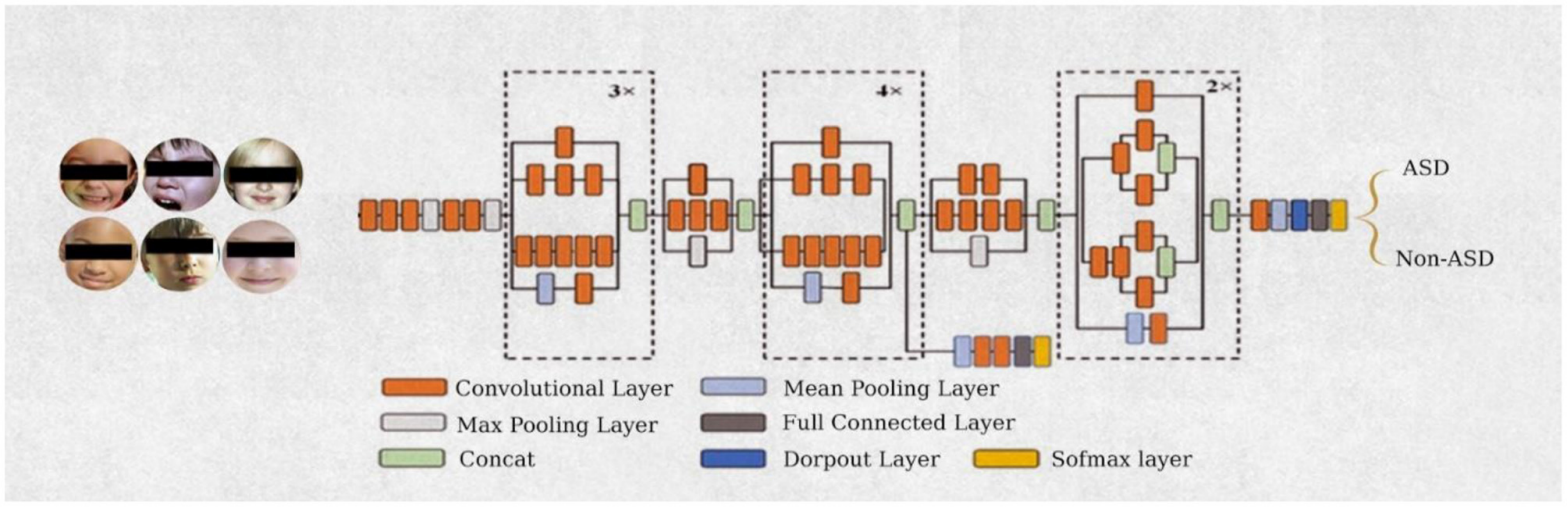
Architecture of inception-V3 network.

Google presented the Inception-V3 pre-trained model. It includes symmetrical and asymmetrical construction blocks, convolutional layers, max and average pooling, concatenations, dropouts, and fully linked layers. Applications of batch normalization in activation layers are typical. The inception-V3 network is the inception block. The inception-V3 model separates layers, and rather than processing via a single layer, it utilizes the input from the preceding layer to execute four distinct processes concurrently, subsequently concatenating the outputs from all these various levels. The 5 × 5 convolution is replaced with two 3 × 3 convolutions in the Inception-V3 architecture, as shown in Figure 5. Since a 5 × 5 convolution requires 2.78 times more resources than a 3 × 3 convolution, this also improves computing performance by decreasing processing time. Utilizing two 3 × 3 layers instead of a single 5 × 5 layer enhances the architecture's performance.

#### 3.3.2 ResNet50 models

**Figure 6:**
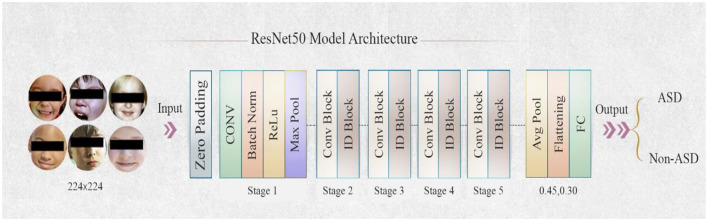
Architecture of ResNet50 model.

Introduced the residual neural network (ResNet) He et al. (33) in 2015. ResNet50 was introduced in 2015 by Microsoft Research for image identification tasks. ResNet indicates that the model has 50 layers. ResNet50 improved training performance by including residual connections between layers, which reduced loss, preserved acquired information, and kept it. An output with a residual link is a convolution of the input and the input itself, or the result of adding both together. Figure 6 illustrates a block diagram of the ResNet50 model's design. Utilized Residual blocks function as shortcuts or skip connections, enabling the model to bypass one or more levels. This mitigates the vanishing gradient issue during training and facilitates the seamless flow of information. ResNet50 key contribution is the invention of the residual block. These leftover blocks facilitate the connection of activations from preceding levels to subsequent layers.

#### 3.3.3 VGG-19 models

**Figure 7:**
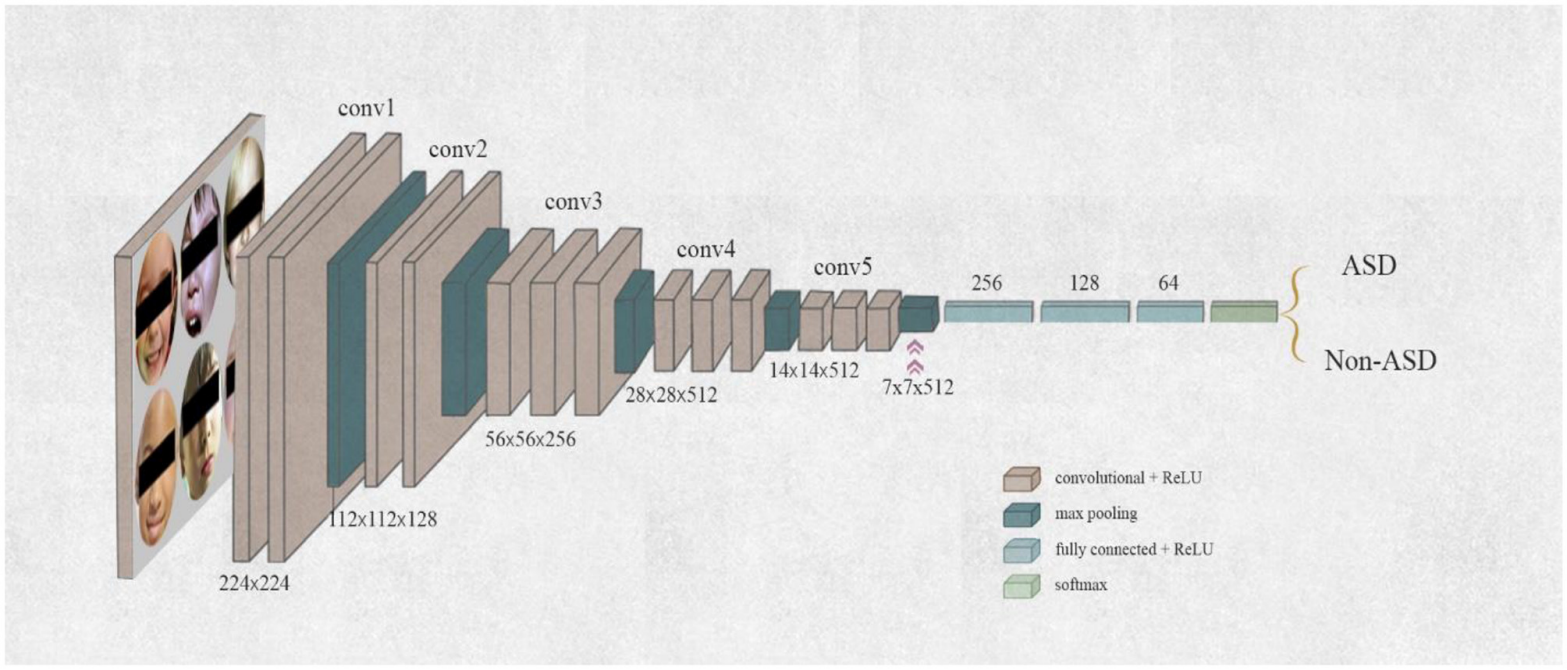
Architecture of VGG-19 model.

The VGG-19 model was introduced by (34). The VGG-19 model for neural networks has 19 weight layers, 16 of which are convolutional layers and 3 of which are fully connected. Its filter size is 3 × 3, and it has a stride and padding of 1 pixel. The diminutive kernel size lowers the parameter count and allows for comprehensive coverage of the whole image. An operation called 2 × 2 max pooling with a stride of 2 is used by the VGG-19 model. With 138 million parameters, this model ranked second in classification and first in positioning in 2014. VGGNet reinforced the notion that CNNs should include a deep layered architecture to facilitate hierarchical interpretation of visual input. Figure 7 illustrates the block model of VGG-19.

### 3.4 Setting of proposed DL models

The DL model is started with pre-trained weights from the ImageNet function, with an input size of ASD image of 224 × 224 × 3, and omitting the top classification layers. The dense layer used sigmoid activation for binary classification objectives. The model used a Stochastic SGD optimizer with a standard learning rate of (0.01), leverages binary cross-entropy for finding performance and loss function, and evaluates performance based on accuracy as the measure. The Training model was used 25 epochs, using early stopping with 5 epochs. The completed model is assessed on the validation set using measures such as accuracy. Classification is performed using Softmax. Table 4 illustrates a schematic representation of the DL model.

**Table 4:** Enhanced parameters for setting the Dl models.

**# No**	**Name**	**Values**
1	Model Architecture	Inception-V3, VGG-19 and ResNet50
2	Image Size	224 × 244 × 3
3	Batch Size	16
4	Learning Rate	0.01
5	Epochs	25
6	Image Rescaling	1./255
7	Optimizer	SGD
8	Pool size	(3,3)
9	Strides	(2,2)
10	Padding	Valid
11	Dencer_layer	512
12	Dropout	0.50
13	Function	Sigmoid

### 3.5 Evaluation metrics

We used critical statistical metrics, including accuracy, precision, and recall, to illustrate our research results. The formulas that are used for the measurement of the DL models are as follows:


(1) $$\begin{array}{l} Accuracy=\frac{TP+TN}{FP+FN+TP+TN}\;\times 100 \end{array}$$

(2) $$\begin{array}{l} F1-score=2*\frac{Precision\;\times \;Recall\;}{Precision+\;Recall\;}\;\times \;100\% \end{array}$$

(3) $$\begin{array}{l} Recall=\;\frac{True\;Positives\;}{True\;Positives+\;False\;positives\;}\;\times \;100\% \end{array}$$

(4) $$\begin{array}{l} Precision=\frac{True\;Negatives}{True\;Negatives+False\;Negatives}\;\times \;100\% \end{array}$$

## 4 Experiment

Training and evaluation of the proposed system were completed on the Kaggle environment platform, which consists of a robust TensorFlow library. We deliberately selected three distinguished pretrained CNNs: Inception-V3, ResNet50, and VGG 19 models, for diagnosis of the autism disorder in children. To use existing best practices and ensure consistency, we selected proven beneficial hyperparameters. Suitable for binary classification tasks, with a learning rate of 0.001, the SGD optimizer, the ReLU activation function, and a maximum of 25 epochs. The specified parameter values were accurately adjusted for all models according to the results of prior cutting-out research, with the objective of attaining optimum training performance for the chosen algorithms. The method was evaluated using a real-time dataset obtained from children with ASD and typically developing children.

### 4.1 Results of ResNet50 models

Table 5 presents the experimental results. The ResNet50 model exhibits significant efficacy in classifying Autistic and Non-Autistic individuals, attaining an overall accuracy of 96%. The ResNet50 model achieves a weighted average precision, recall, and F1-score of 96%, demonstrating consistent performance across both classes. In the Non-autistic class, precision is 98%, indicating that nearly all autistic predictions are accurate, whereas recall is 94%, indicating that some autistic cases are observed. The Autistic class demonstrates a precision of 94%, suggesting the presence of some false positives, while achieving a recall of 98%, indicating that nearly all Non-Autistic cases are identified. The F1-scores of 96% for Autistic individuals and 96% for Non-Autistic individuals indicate a strong balance in classification performance. The results indicate the model's effectiveness; however, lower enhancements in Non_Autistic precision may be realized through further data augmentation or fine-tuning. ResNet50 model demonstrates significant reliability for the classification of images related to autism, as proved by this evaluation.

**Table 5:** Validation results of ResNet50 model.

**Model**	**Precision (%)**	**Recall (%)**	**F1 score (%)**	**Support**
Non_Autistic	98	94	96	294
Autistic	94	98	96	294
Accuracy %	96			
Weighted Avg.	96	96	96	588

**Figure 8:**
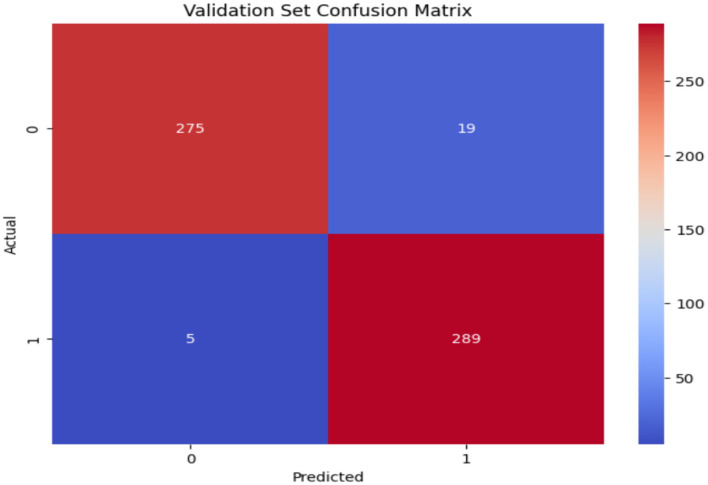
Confusion matrix of ResNet50 model.

Figure 8 presents the classification of the validation set of the ResNet50 model. The classification model's performance on the validation set was assessed through a confusion matrix. The model accurately identified 275 TN and 289 TP, exhibiting minimal FP. The model demonstrates high accuracy, minimal FP, and effective class differentiation, rendering it reliable for classification tasks.

### 4.2 Results of Inception-V3

The Inception-V3 model exhibits exceptional accuracy and robust classification capabilities in detecting ASD, as shown in Table 6. The Inception-V3 model demonstrates high accuracy and strong classification performance in the detection of ASD, achieving an overall accuracy of 98%. The system demonstrates a precision of 98% in identifying non-autistic cases, accompanied by an F1 score of 98%. The precision for Autistic cases is 97%, with a recall of 98% and an F1 score of 98%. This balanced performance minimizes misclassifications, rendering it appropriate for real-world applications in the identification of ASD with confidence and precision. The results demonstrate that the model effectively classifies target classes while maintaining a low misclassification rate, thereby rendering it suitable for real-world applications in the identification of ASD with high confidence and precision.

**Table 6:** Validation results of Inception-V3 model.

**Model**	**Precision (%)**	**Recall (%)**	**F1 score (%)**	**Support**
Non_Autistic	98	97	98	294
Autistic	97	98	98	294
Accuracy %	98			
Weighted Avg.	98	98	98	588

**Figure 9:**
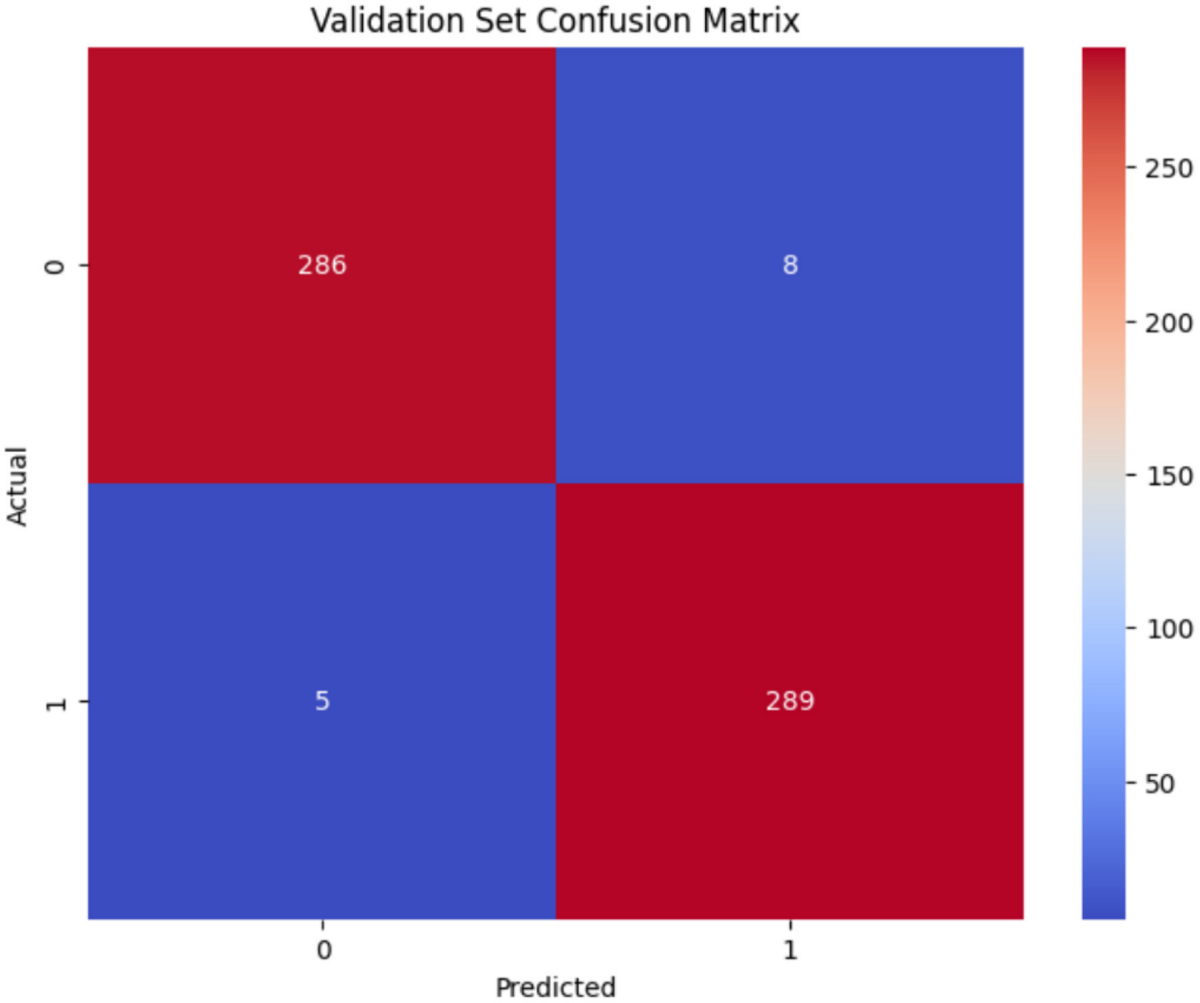
Confusion matrix of Inception-V3 model.

Figure 9 presents the confusion matrix for the Inception-V3 model during the validation stage. The model demonstrated enhanced classification performance. The Inception-V3 model exhibited robust classification performance, successfully predicting 286 non-autistic and 289 Autistic cases from a total of 588 samples. The model exhibited minimal misclassifications, recording 8 false positives (FP) and 5 false negatives (FN), which suggests strong recall and precision. The model demonstrated reliability and balanced performance, though there remains potential for improvement in minimizing misclassification rates.

### 4.3 Result of VGG-19

The VGG19 model demonstrates high precision, recall, and F1-score in the classification of ASD, exhibiting minimal FP and TN, as shown in Table 7. It demonstrates strong performance in the Autistic and Non-Autistic classes, as indicated by precision, recall, and F1-score metrics. The model reveals a 97% accuracy rate, suggesting its appropriateness for clinical ASD detection, with opportunities for enhancement via refined training strategies.

**Table 7:** Validation results of VGG-19 model.

**Model**	**Precision (%)**	**Recall (%)**	**F1 score (%)**	**Support**
Non_Autistic	98	97	97	294
Autistic	97	98	97	294
Accuracy %	97			
Weighted Avg.	97	97	97	588

**Figure 10:**
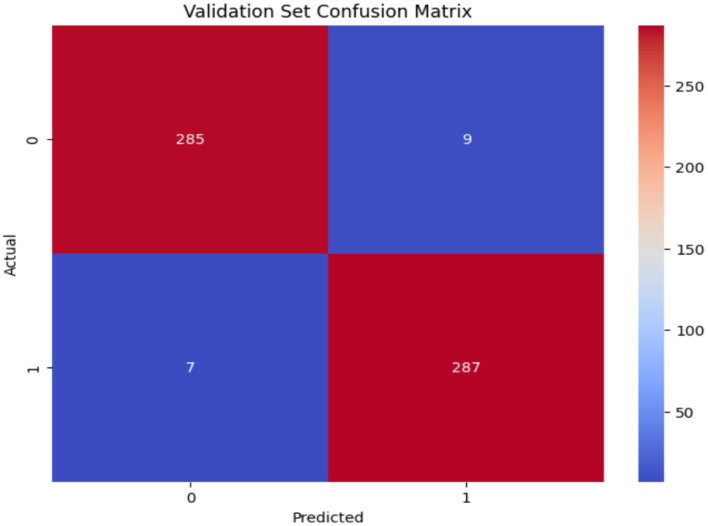
Confusion matrix of ResNet50 model.

The confusion matrix of VGG19 is shown in Figure 10. The VGG19 model demonstrated robust performance on the validation dataset, with 285 TN accurately identifying the Non_Autistic class and 287 TP correctly identifying the Autistic class. There are just 9 FP as misclassifcation as Autistic when the true class is Non_Autistic, and 7 FN misclassifying as Non_Autistic when the true class is Autistic, resulting in a minimal total count of misclassifications.

### 4.4 Performance of the ASD system based on DL models

The ASD detection system, using deep learning models, has impressive accuracy rates of 98% in training and validation, distinguishing between non-autistic and Autistic patients. The model's robust convergence and consistent validation outcomes demonstrate its proficiency in generalizing novel data, making it a valuable early identification tool.

**Figure 11:**
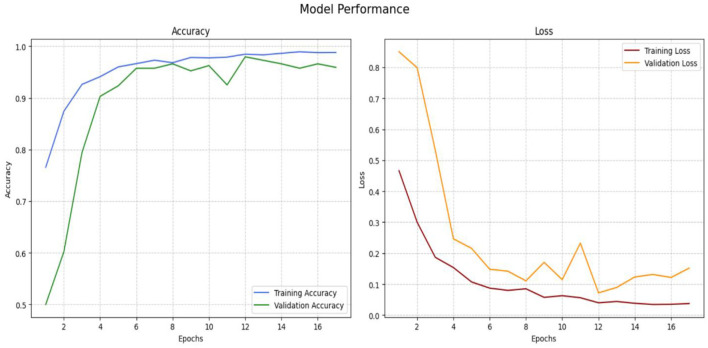
Performance of ResNet50 model.

Figure 11 shows the accuracy and loss of the ResNet50 system, with a *y*-axis representing data classification accuracy. The validation system improved accuracy from 0.5000 to 0.9592 during the validation phase, with an exceptional enhancement to 25 epochs. Training losses were quantified using a categorical cross-entropy function, with validation losses decreasing from 0.5 to 0.01 after 25 epochs.

**Figure 12:**
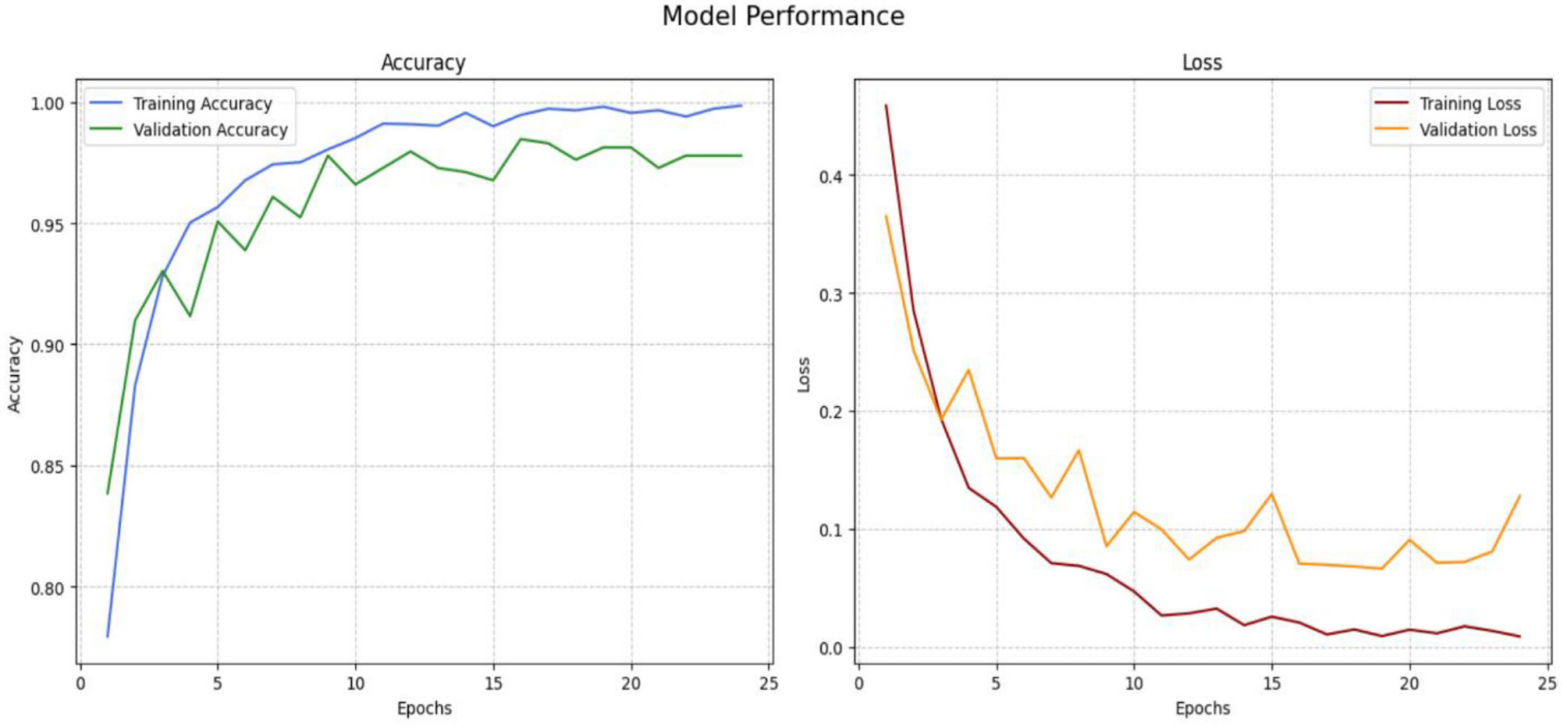
Performance of Inception-V3 model.

The performance of the Inception-V3 model is seen in Figure 12 for both training and validation. We use categorical entropy loss and the SGD optimizer, executing for 25 epochs. During the training phase, the loss value diminishes from 0.7265 to 0.0076 until 25 epochs. The training accuracy is increasing gradually from 0.4844 to 0.9992 epoch 2 to 25. While validation accuracy improves from 0.8384 to 0.9779 throughout 25 epochs. This illustrates the model's capacity to learn and adjust according to input data. From epoch 3 to epoch 25, the model's performance improved progressively, exhibiting enhanced accuracy and less loss. Attaining a accuracy of 0.98 is a significant achievement.

**Figure 13:**
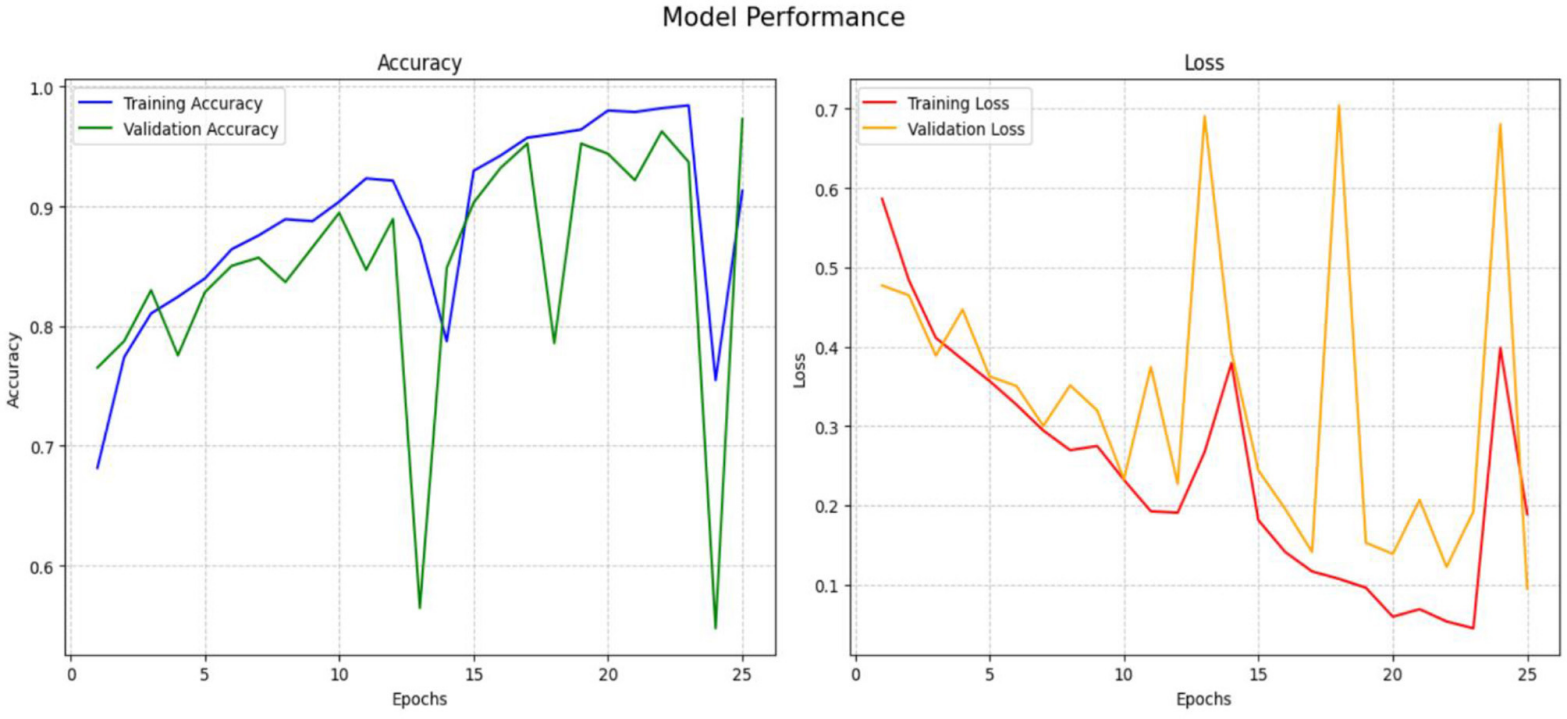
Performance of VGG-19 model.

Figure 13 illustrates the accuracy and loss performance of VGG19. During training epochs 2 to 23, the model's accuracy increases to above 0.9854; however, there is a significant decline in accuracy from epochs 24–25. The validation accuracy reaches a maximum of 0.97 in the latter epochs, namely at epoch 25, demonstrating the model's effective recognition of the dataset's intrinsic patterns. The model's validation accuracy on unfamiliar data increases from 0.7653 in the opening epoch to an impressive 0.9728 at the conclusion of the 25th epoch. The validation loss consistently decreased throughout the preceding period, ultimately reaching a minimum of 0.0947.

## 5 Discussion

Individuals with ASD have difficulties in social interaction, communication, and conduct, as well as a variety of other neurological issues. Timely identification is crucial for mitigating the detrimental effects of this disease by implementing specialized instruction in schools and rehabilitation facilities. The research examined DL algorithms for the detection of autism spectrum disorder, emphasizing its efficacy in differentiating between persons with and without the condition. Current research primarily focuses on functional discoveries for categorization tasks, often leading to decreased accuracy. Our suggested methodology redirects attention to using structural information within facial expression data. Utilizing DL approaches, namely Inception-V3, and optimizing hyperparameters within this framework, we seek to address the shortcomings of existing procedures while augmenting generalization capacities and enhancing classification accuracy. This motivation stems from the recognition of the underutilized potential of facial expressions in children with ASD and typically developing children, along with the conviction that harnessing this information can lead to more effective classification models for diverse neurological conditions, thereby advancing the field and improving patient outcomes.

The potential threat we faced in this work is that data bias may undermine the model's generalizability, especially if the dataset lacks sufficient demographic diversity or exhibits class imbalance between autistic and non-autistic images. We have employed the augmentation method to address this issue, utilizing augmentation, early stopping, and transfer learning regularization techniques to mitigate overfitting. Including images from the same subject or session in several data splits might cause dataset leakage. This threat raises interpretability issues since it may be unclear which image features the models prioritize in their decision-making process. This pre-processing improved DL models, namely ResNet50, Inception-V3, and VGG-19, and removed the threat, achieving high accuracy. Finally, the DL models were examined by using accuracy and confusion matrices.

This approach used the augmentation technique to enhance the deep learning model for diagnosing ASD with outstanding performance. Employing ResNet50, Inception-V3, and VGG-19 models resulted in substantial improvements in diagnostic accuracy, with an exceptional 98% accuracy in differentiating between ASD and control subjects on the standard dataset. The results of ResNet50 scored 96% in terms of accuracy, and VGG-19 achieved an accuracy of 97%. The efficacy of this strategy is further substantiated by criteria such as accuracy, underscoring its potential to improve autism outcomes. The results have significant implications for ASD diagnosis in clinical settings, enabling more informed decisions, earlier identification and intervention, and improved outcomes for individuals and their families. Advanced algorithms may optimize the diagnosis process, thereby decreasing wait times and lowering the urgency on the healthcare system. Additional study and validation on more extensive datasets are required to comprehensively evaluate their therapeutic value and effect.

**Figure 14:**
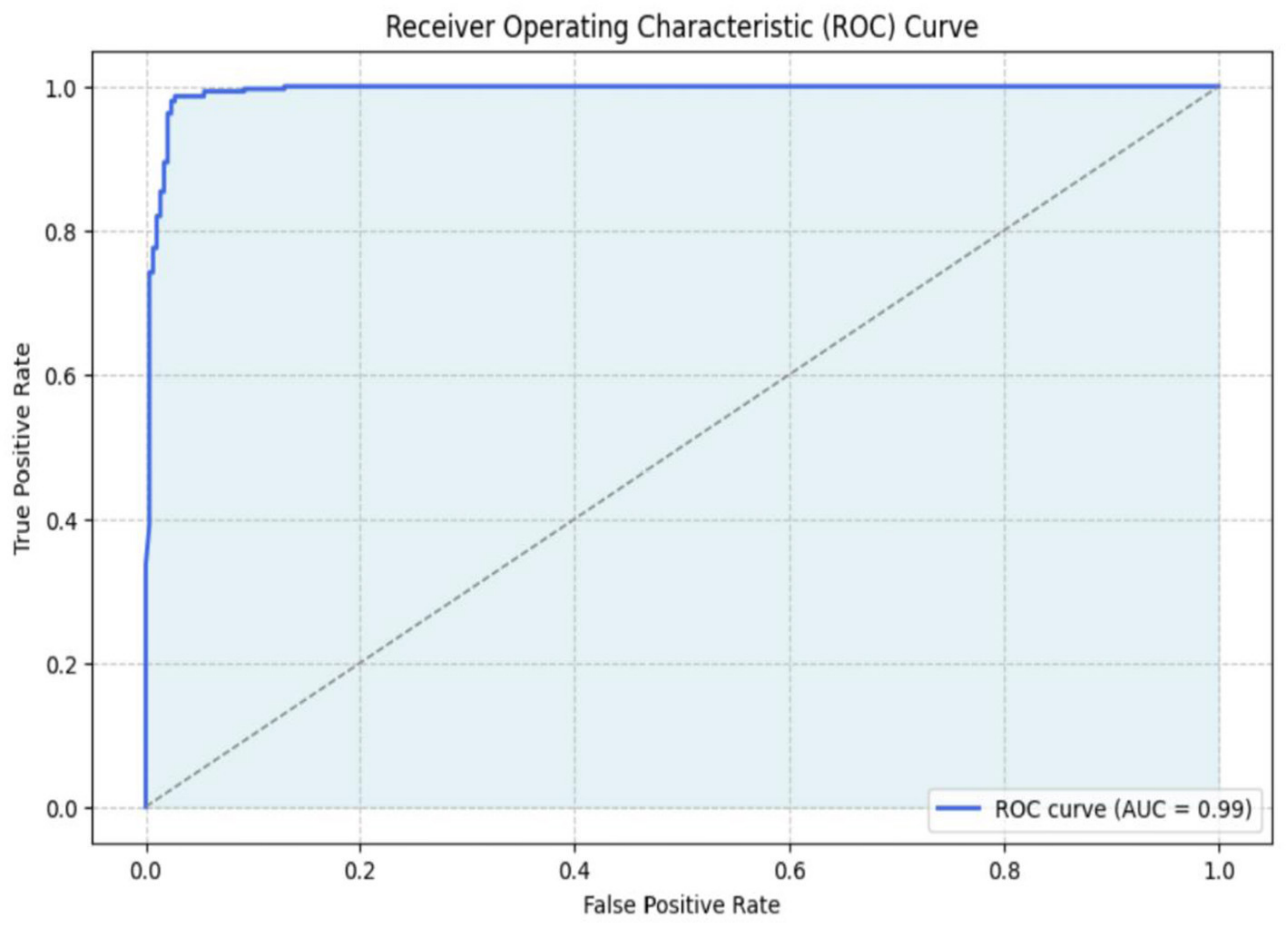
ROC of Inception-V3 model.

The AUC, or area under the curve, signifies that a higher AUC correlates with an increased probability of precise prediction. Figure 14 illustrates the ROC curve of the optimal methodology. The Inception-V3 model has superior accuracy and AUC of 99% across all three methodologies.

**Figure 15:**
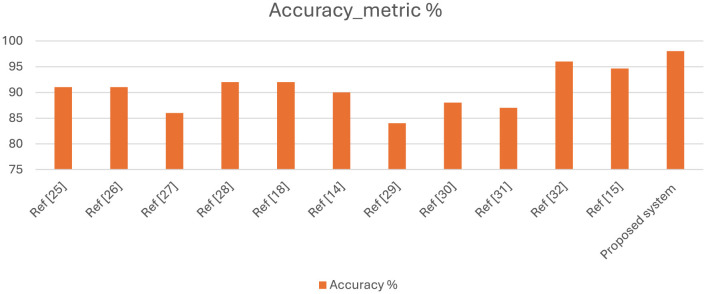
Performance of proposed system compared with different existing ASD systems.

Numerous studies have been conducted specifically in diagnosing ASD based on the image expression of children. Most authors used the same standard dataset, available on Kaggle, which contains 2,940 images for applying different automatic classification approaches to diagnose ASD based on facial images, thereby enhancing accuracy. Prior studies indicate that suboptimal image quality in the training dataset significantly affects the accuracy of model results. One of the biggest challenges faced by the researchers is that images of children's faces frequently exhibit noise, low resolution, misalignment, and various other issues. Several researchers focus on optimizing models or hyperparameter sets, yet they often fail to achieve significant improvements in accuracy. Table 8 presents a comparison of the results from the latest studies in this field. In our research, we have improved the hyperparameters of the proposed DL model, and we have achieved 98% accuracy using the same dataset. Figure 15 compares our system's results with those of other approaches, highlighting the superior accuracy of our proposed strategy.

**Table 8:** Results of existing developing ASD systems with our results.

**References**	**Approach**	**Used datasets**	**Accuracy (%)**
Rashid and Shaker (25)	Xception	Same dataset	91
Alsaade and Alzahrani (26)	Xception		91
Sridurga et al. (27)	Xception		86
Rabbi et al. (28)	CNN		92
Alkahtani et al. (18)	MobileNetV2		92
Akter et al. (14)	MobileNet-V1		90
Gaddala et al. (29)	VGG16 & 19		84
Singh et al. (30)	MobileNet		88
Ghazal et al. (31)	AlexNet		87
Hosseini et al. (32)	MobileNet		94.64
Elshoky et al. (15)	ML		96
MobileNetV2	MobileNetV2		92
Proposed system			98

## 6 Conclusion

Diagnosing at an early stage is essential for administering successful treatment, particularly given the very low incidence of autism in children. The DL algorithms were used for ASD detection, often concentrating only on diagnosis. Moreover, current systems may have difficulties in scaling efficiently due to belief in manual and expertise-dependent procedures, impeding their capacity to satisfy the growing demand for autism evaluation and diagnosis. To tackle these issues, we have developed an efficient DL model„ namely ResNet50, Inception-V3, and VGG-19, implemented to predict and diagnose ASD. Pre-processing techniques, including resizing, rescaling, and augmentation, were used to enhance model performance, which may further elevate accuracy. Our classifiers achieved exceptional accuracies of 96%, 98%, and 97% for ASD, expression prediction, respectively. This illustrates their ability to precisely distinguish children's psychological states and facial expressions. We developed ASD system-based DL model to assess children's expressions and diagnose ASD. This study has significant effects for real-time ASD screening, potentially transforming the diagnosis process.

## Data Availability

Publicly available datasets were analyzed in this study. This data can be found here: https://www.kaggle.com/datasets/cihan063/autism-image-data.
